# Risk Factors and Outcomes of AKI after LAAC Operation: A Single-Center Observational Study from Mainland China

**DOI:** 10.31083/j.rcm2309306

**Published:** 2022-09-09

**Authors:** Lei Zhang, Jiarui Xu, Xiaoye Li, Xiaochun Zhang, Wenzhi Pan, Lihua Guan, Xiaoqiang Ding, Daxin Zhou, Junbo Ge

**Affiliations:** ^1^Department of Cardiology, Zhongshan Hospital, Fudan University, 200032 Shanghai, China; ^2^National Clinical Research Center for Interventional Medicine, 200032 Shanghai, China; ^3^Shanghai Clinical Research Center for Interventional Medicine, 200032 Shanghai, China; ^4^Department of Nephrology, Zhongshan Hospital, Fudan University, 200032 Shanghai, China; ^5^Shanghai Institute of Kidney Disease and Dialysis, 200032 Shanghai, China; ^6^Department of Pharmacy, Zhongshan Hospital, Fudan University, 200032 Shanghai, China

**Keywords:** atrial fibrillation, left atrial appendage closure, acute kidney injury

## Abstract

**Background:**

This study aimed to investigate the predictors and prognosis of acute kidney injury (AKI) occurrence among Chinese patients following left atrial appendage closure (LAAC).

**Methods:**

We retrospectively enrolled 512 consecutive patients who underwent LAAC between January 2014 and December 2019. AKI was clinically defined according to the Kidney Disease Improving Global Outcomes serum creatinine criteria. Major adverse cardiovascular events were defined as the composite of all-cause mortality, readmission due to heart failure, cardiac surgery, systemic embolism, or bleeding events.

**Results:**

The incidence of AKI was 5.3% and was highest in patients with chronic kidney disease (CKD) stages 4–5 (25.0%), followed by those with CKD stages 3a–3b (9.1%), and those with CKD stages 1–2 or without CKD (3.9% only). Multivariate logistic regression showed that lower body mass index (odds ratio [OR] = 0.889; 95% confidence interval [CI], 0.803–0.986; *p* = 0.017), hypertension (OR = 5.577; 95% CI, 1.267–24.558; *p* = 0.023), and CKD stages 4–5 (OR = 6.729; 95% CI, 1.566–28.923; *p *= 0.010) were independent risk factors for AKI development after LAAC. AKI after LAAC was associated with 3-year major adverse cardiovascular events (33.3% vs. 7.5%, *p <* 0.001) and all-cause mortality (11.1% vs. 0.9%, *p *< 0.001) compared to that in the non-AKI group.

**Conclusions:**

AKI is relatively common after LAAC in patients with a baseline impaired glomerular filtration rate. Moreover, AKI after LAAC is mainly related to increased midterm mortality and morbidity, which require more strategies for prevention and treatment.

## 1. Introduction

Acute kidney injury (AKI) is commonly regarded as a complication of many cardiac interventions such as percutaneous coronary intervention (PCI) [1] and transcatheter aortic valve replacement (TAVR) [2]. Left atrial appendage closure (LAAC) is the recommended alternative for patients with nonvalvular atrial fibrillation (NVAF) who cannot tolerate long-term oral anticoagulation (OAC) treatment [3, 4]. Although LAAC is considered a safe and effective procedure, similar to other cardiac interventions that require contrast media, patients who undergo LAAC are also at risk of AKI. Recent studies have shown that in clinical practice, AKI after LAAC has implicit adverse effects [5, 6]. However, knowledge on AKI after LAAC is insufficient, and data regarding this setting remain inadequate. Furthermore, patients who undergo LAAC operation are mostly the elderly, with accompanying kidney dysfunction, diabetes, and chronic cardiopulmonary disease, all of which are risk factors for AKI [7]. Therefore, this study aimed to investigate the predictors and prognosis of AKI among Chinese patients following the LAAC procedure.

## 2. Materials and Methods

### 2.1 Study Population

**Fig. 1.**
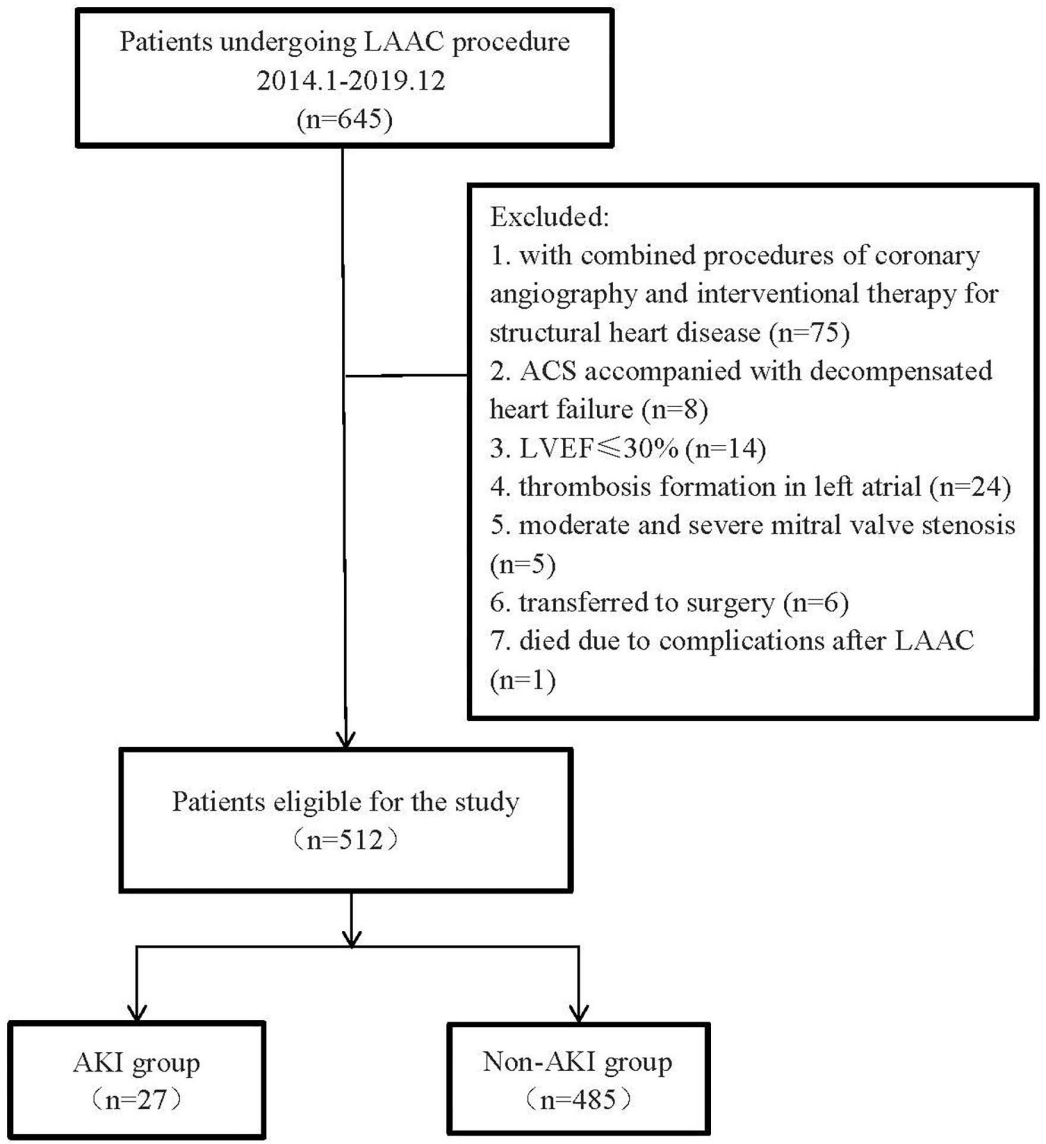
**The flowchart of the study**. AKI, acute kidney injury.

This observational study collected the clinical data of consecutive patients who underwent LAAC between January 2014 and December 2019. The inclusion criteria were as follows: diagnosis of paroxysmal or persistent NVAF, thrombosis risk score (${\mathrm{CHA}}_{2}$${\mathrm{DS}}_{2}$-VASc) ≥2, complications with a high bleeding risk (HAS-BLED) score ≥3, and ineligibility for long-term OAC. The exclusion criteria were as follows: (1) concomitant coronary angiography and interventional therapy for structural heart disease (e.g., heart defect closure, transcatheter aortic valve replacement, pacemaker implantation), (2) acute coronary syndrome accompanied by decompensated heart failure, (3) left ventricular ejection fraction (LVEF) ≤30%, (4) thrombosis formation in the left atrium, and (5) moderate and severe mitral valve stenosis. In addition, patients who were transferred to surgery or died due to complications of LAAC procedures were also excluded (Fig. 1).

Baseline characteristics, including demographics and complications, LAAC indications, ${\mathrm{CHA}}_{2}$${\mathrm{DS}}_{2}$-VASc and HAS-BLED risk scores, antithrombotic medications, procedural details, and clinical and echocardiographic follow-up events, were collected through the electronic medical system records, and data were retrospectively analyzed.

### 2.2 Device and Implanting Procedure

The LAAC device was implanted using a catheter-based delivery system after atrial septal puncture. Generally, closure was performed under general anesthesia and guided by fluoroscopy combined with transesophageal echocardiography (TEE). Left atrial appendage (LAA) angiography was performed 30° right anterior oblique (RAO) and 20° caudal (CAU) to measure the LAA orifice. The device was delivered through a catheter and expanded to close the LAA opening. The device was released after confirming its stability without a large residual shunt (≥5 mm). Two types of occluders were used in our center: the Watchman device (Boston Scientific, MA, USA) and a LAmbre device (Lifetech Scientific [Shenzhen] Co. Ltd, Shenzhen, China). The device selection was mainly based on the operator’s discretion; however, the LAmbre occluder was mostly used in patients with complicated LAA morphology. Heparin (80–100 IU/kg of body weight) was administered after atrial septal puncture, with an activated clotting time ranging from 250 s to 300 s throughout the entire operation.

### 2.3 Definition of AKI and CKD

AKI was defined based on the change in serum creatinine (SCr) concentration from the baseline to the peak value measured within 7 days after LAAC. According to the Kidney Disease Improving Global Outcomes (KDIGO) guidelines for AKI [8], an increase in SCr concentration by ≥0.3 mg/dL within 48 h or an increase in SCr concentration to ≥1.5 times compared with that of baseline within 7 days was considered a clinically established diagnosis of AKI.

CKD was diagnosed and staged according to the KDIGO criteria [9]: (1) regardless of a decreased glomerular filtration rate (GFR) [10] or kidney damage lasting >3 months as defined by structural or functional abnormalities of the kidney and (2) GFR <60 mL/min/1.73 $m^{2}$ that continued for >3 months, regardless of kidney damage.

### 2.4 Prevention of AKI in High-Risk Patients

Patients with CKD at baseline received prophylactic intravenous hydration with isotonic saline 12 h before LAAC and continued for at least 24 h afterwards. Renal function (SCr level and estimate glomerular filtration rate, eGFR) was monitored daily until the patient was discharged. The physician team will decide whether to use glutathione or bicarbonate, if necessary. Attention was paid on perioperative complications, especially the need for hemodialysis, during hospitalization.

### 2.5 Postoperative Anticoagulation Strategy

Patient received warfarin with an international normalized ratio (INR) between 2.0–2.5 after LAAC and novel oral anticoagulants (NOACs) were prescribed for those with contraindications to warfarin anticoagulation. TEE was completed 45 days after surgery to determine (>5 mm) or device-related thrombus (DRT). Warfarin or NOAC therapy was discontinued after confirming the absence of DRT and switched to dual antiplatelet therapy using aspirin and clopidogrel for another 4.5 months. Subsequently, long-term single antiplatelet therapy was maintained. In patients with a high risk of bleeding, short-term (≤1 year) antithrombotic therapy was considered an alternative.

### 2.6 In-Hospital and Out-of-Hospital Follow-Up

Prior to discharge, TTE was performed to rule out cardiac effusion, and SCr was tested to ensure no remarkable deterioration of renal function. The patients underwent TEE evaluation of residual leak and DRT at 6 weeks and 6 months after the procedure. Follow-up was performed during outpatient clinical visits or via telephone call. The follow up time ranged from 2 to 48 months. The comparison of the primary efficacy endpoint was defined as major adverse cardiovascular events (MACE) in terms of all-cause mortality, readmission due to heart failure, cardiac surgery, and systemic embolism, and the primary safety endpoint was defined as major periprocedural complications such as major bleedings during follow-up visits [11].

### 2.7 Statistical Analysis

Continuous variables were presented as mean ± standard deviation (SD), and categorical variables as frequencies and percentages (%). Chi-square or Fisher’s exact tests were performed to compare qualitative variables and Student’s *t*-test for numerical variables. In addition, the Kaplan–Meier curve was used to compare all-cause mortality between the two groups. Multivariate analysis was used to assess the independent predictors of all-cause 1-year mortality. A binary logistic multivariate regression model including clinically relevant baseline parameters such as age, sex, LVEF, SCr concentration, and other parameters (*p <* 0.1 in univariate analysis) was established. All statistical analyses were performed using the SPSS software (version 25.0.0.1; IBM Corporation, Somers, NY, USA). Statistical significance was set at *p *< 0.05.

## 3. Results

### 3.1 Basic Characteristics

A total of 512 eligible patients (mean age 69 years) were enrolled in this study. Twenty-seven (5.3%) patients developed AKI according to the KDIGO SCr criteria. The baseline clinical characteristics of the study population are shown in Table 1. Significant differences in sex, age, or body mass index (BMI) were not observed between the AKI and non-AKI groups. Patients with AKI were more likely to have comorbid hypertension than that of non-AKI patients (92.6% vs. 67.2%, *p* = 0.006). Patients in the AKI group tend to have a higher CKD stage (CKD stages 3a–3b, 29.6%, and CKD stages 4–5, 11.1%, and only 16.5% and 1.9%, respectively, in the non-AKI group). The HAS-BLED score was significantly higher in the AKI group (3.3 ± 1.1 vs. 2.9 ± 1.1, *p* = 0.028) (Table 1).

**Table 1.** **Basic characteristics of AKI vs. non-AKI group**.

		All	non-AKI group	AKI group	*p* value
		n = 512	n = 485	n = 27	
Male [n (%)]		308 (60.2%)	296 (61.0%)	12 (44.4%)	0.087
Age (y)		69 ± 9	69 ± 9	71 ± 10	0.151
Elderly [n (%)]		341 (66.6%)	342 (70.5%)	20 (74.1%)	0.693
BMI (${\mathrm{kg/m}}^{2}$)		24.6 ± 5.7	24.7 ± 5.7	22.8 ± 5.8	0.094
Hypertension [n (%)]		351 (68.6%)	326 (67.2%)	25 (92.6%)	0.006
Diabetes [n (%)]		112 (21.9%)	103 (21.2%)	9 (33.3%)	0.139
Stroke [n (%)]		210 (41.1%)	200 (41.3%)	10 (37.0%)	0.660
Vascular disease [n (%)]		48 (9.4%)	44 (9.1%)	4 (14.8%)	0.319
Echocardiography					
	LVEF (%)	64 ± 19	64 ± 19	63 ± 17	0.785
	LAA Diameter (mm)	21 ± 4	21 ± 4	21 ± 6	0.838
	LAA Length (mm)	25 ± 6	26 ± 6	24 ± 5	0.160
SCr (μmol/L)		81 [69, 94]	81 [69, 94]	85 [73, 97]	0.698
eGFR (mL/min/1.73 $m^{2}$)		75.6 ± 17.9	75.8 ± 17.3	70.9 ± 26.2	0.347
CKD stage					0.001
	no CKD/stage 1–2 [n (%)]	412 (80.5%)	396 (81.6%)	16 (59.3%)	
	stage 3a–3b [n (%)]	88 (17.2%)	80 (16.5%)	8 (29.6%)	
	stage 4–5 [n (%)]	12 (2.3%)	9 (1.9%)	3 (11.1%)	
hs-CRP (mg/L)		0.7 [0.3, 1.9]	0.7 [0.3, 1.9]	0.7 [0.3, 2.6]	0.823
D-dimer (mg/L)		0.27 [0.19,0.74]	0.26 [0.19, 0.72]	0.60 [0.19, 0.97]	0.120
${\mathrm{CHA}}_{2}$${\mathrm{DS}}_{2}$-Vasc		3.5 ± 1.4	3.5 ± 1.4	4.0 ± 1.7	0.079
HAS-BLED		2.9 ± 1.1	2.9 ± 1.1	3.3 ± 1.1	0.028

AKI, acute kidney injury; BMI, body mass index; LVEF, left ventricular ejection fraction; LAA, left atrial appendage; SCr, serum creatinine; eGFR, estimated glomerular filtration rate; CKD, chronic kidney disease; hs-CRP, highly sensitive C-reactive protein.

### 3.2 Procedural Characteristics and Outcomes

Significant differences in LAA morphology, device type, device size, or antithrombotic therapy between the AKI and non-AKI groups were not found. The contrast volume administered during the procedure (154 ± 23 mL vs. 145 ± 22 mL; *p* = 0.051) and the contrast volume-to-glomerular filtration (CV/GFR) ratio were higher in the AKI group (2.3 [1.8, 2.7] vs. 1.9 [1.6, 2.3], *p* = 0.055), but the difference was not significant (Table 2).

**Table 2.** **Procedural characteristics and outcomes of AKI vs. non-AKI group**.

		All	non-AKI group	AKI group	*p* value
		n = 512	n = 485	n = 27	
Procedure data					
LAA morphology					0.830
	cactus [n (%)]	33 (6.4%)	32 (6.6%)	1 (3.7%)	
	cauliflower [n (%)]	359 (70.1%)	340 (70.1%)	19 (70.4%)	
	chickenwing [n (%)]	82 (16.0%)	78 (16.1%)	4 (14.8%)	
	windsock [n (%)]	38 (7.4%)	35 (7.2%)	3 (11.1%)	
Landing zone diameter (mm)		26 ± 5	26 ± 5	23 ± 4	0.069
Device					0.455
	Watchman [n (%)]	366 (71.5% )	345 (71.1%)	21 (77.8%)	
	Lambre [n (%)]	146 (28.5%)	140 (28.9%)	6 (22.2%)	
Device size (mm)					
Watchman					0.513
	21 [n (%)]	21 (5.7%)	19 (5.5%)	2 (9.5%)	
	24 [n (%)]	41 (11.2%)	39 (11.3%)	2 (9.5%)	
	27 [n (%)]	104 (28.4%)	96 (28.7%)	8 (38.1%)	
	30 [n (%)]	88 (24.0%)	83 (24.8%)	5 (23.8%)	
	33 [n (%)]	112 (30.6%)	108 (32.2%)	4 (19.0%)	
Lambre					
	Umbrella size (mm)	27.1 ± 6.1	27.1 ± 6.1	27.7 ± 6.0	0.960
	Cover size (mm)	34.8 ± 4.4	34.8 ± 4.4	34.7 ± 4.8	0.824
Contrast volume (mL)		146 ± 22	145 ± 22	154 ± 23	0.051
CV/GFR ratio		1.9 [1.6, 2.3]	1.9 [1.6, 2.3]	2.3 [1.8, 2.7]	0.055
Antithrombotic regimen					
	VKA [n (%)]	48 (9.4%)	46 (9.5%)	2 (7.4%)	0.741
	NOAC [n (%)]	392 (76.6%)	370 (76.3%)	22 (81.5%)	0.532
	DAPT [n (%)]	72 (14.1%)	69 (14.2%)	3 (11.1%)	0.288
Outcomes					
DRT [n (%)]		16 (3.1%)	13 (2.7%)	3 (11.1%)	0.012
Residual shunt					0.237
	None [n (%)]	368 (71.9%)	346 (71.3%)	22 (81.5%)	
	1–5 [n (%)]	138 (26.9%)	134 (27.6%)	4 (14.8%)	
	≥5 [n (%)]	6 (1.2%)	5 (1.0%)	1 (3.7%)	
3-year MACE					<0.001
	HF rehospitalization [n (%)]	12 (2.3%)	8 (1.6%)	4 (14.8%)	
	Stroke/Ischemic events [n (%)]	16 (3.1%)	14 (2.9%)	2 (7.4%)	
	Cardiac surgery [n (%)]	5 (1.0%)	4 (0.8%)	1 (3.7%)	
	Major bleeding [n (%)]	6 (1.2%)	5 (1.0%)	1 (3.7%)	
	Cardiac death [n (%)]	3 (0.6%)	1 (0.2%)	2 (7.4%)	
3-year Mortality [n (%)]		6 (1.2%)	3 (0.6%)	3 (11.1%)	<0.001

AKI, acute kidney injury; LAA, left atrial appendage; CV/GFR, the contrast volume-to-glomerular filtration; VKA, vitamin K antagonists; NOAC, novel oral anticoagulant; DAPT, dual antiplatelet therapy; DRT, device related thrombi; MACE, major adverse cardiac event; HF, heart failure.

The DRT ratio was significantly higher in the AKI group than that in the non-AKI group (11.1% vs. 2.7%, *p* = 0.012). No significant difference in residual shunt was observed between the two groups. The incidence of different types of long-term MACE after the procedure was significantly higher in the AKI group than that in the non-AKI group. Long-term all-cause mortality after the procedure was significantly higher in the AKI group than that in the non-AKI group (11.1% vs. 0.6%, *p *< 0.001).

### 3.3 Logistic Regression Analyses of the AKI Risk Factors

**Fig. 2.**
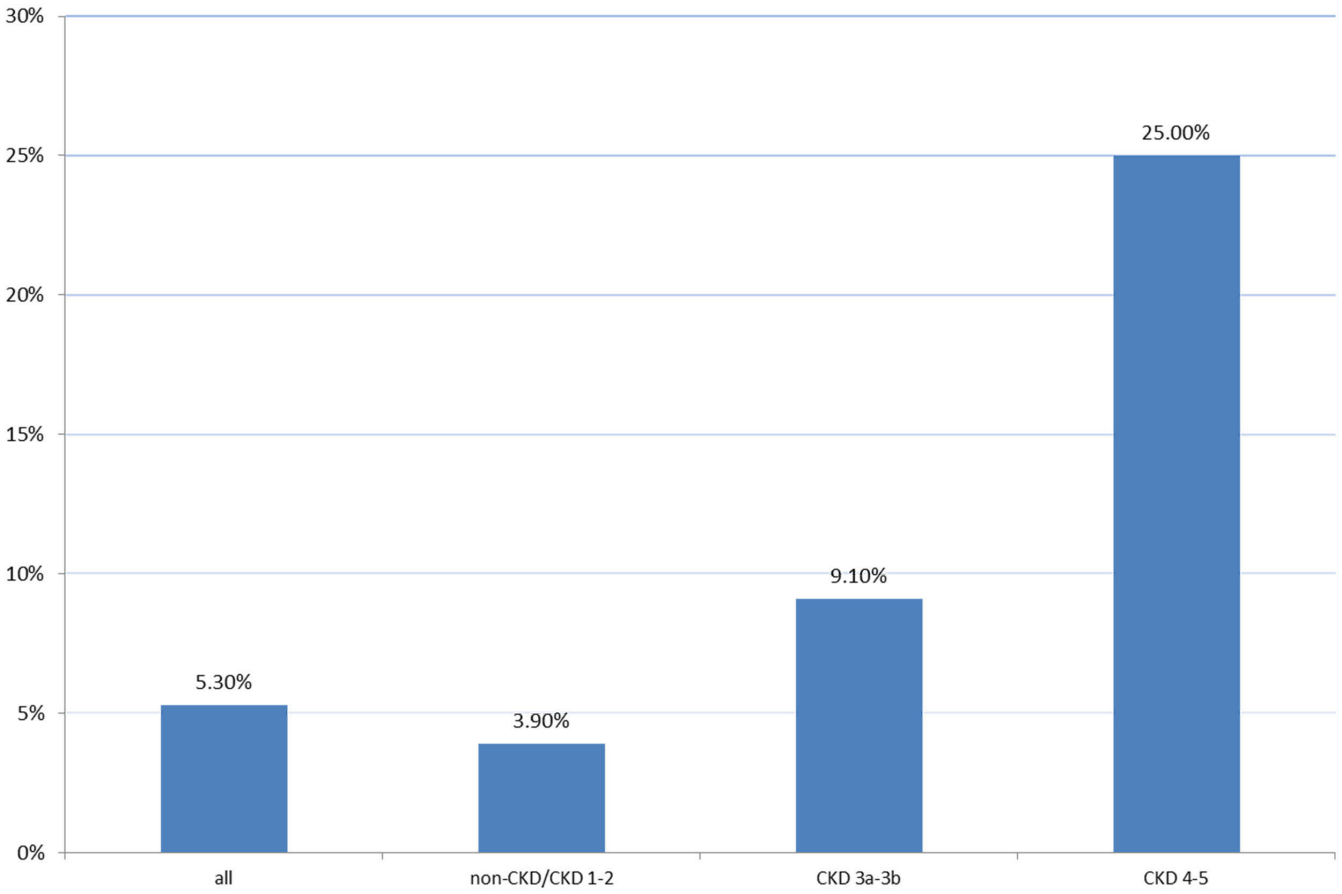
**AKI incidence between different CKD stages**. AKI, acute kidney injury; CKD, chronic kidney disease.

The AKI incidence was highest in patients with CKD stages 4–5 (25.0%), followed by those with CKD stages 3a–3b (9.1%), and those with CKD stages 1–2 or without CKD (only 3.9%) (Fig. 2).

Variables with *p *< 0.10 were included in the univariate regression model found in Table 1. Univariate regression analysis showed that lower BMI, hypertension, CKD stages 3a–3b and 4–5, and HAS-BLED score were risk factors for the development of AKI (*p *< 0.05). Multivariate logistic regression analysis showed that lower BMI (OR = 0.889; 95% confidence interval [CI], 0.803–0.986; *p* = 0.017), hypertension (OR = 5.577; 95% CI, 1.267–24.558; *p* = 0.023), and CKD stages 4–5 (OR = 6.729; 95% CI, 1.566–28.923; *p* = 0.010) were risk factors for the development of AKI after LAAC (Table 3).

**Table 3.** **Logistic regression analysis of risk factors for AKI after LAAC**.

		Univariate		Multivariate	
		OR (95% CI)	*p* value	OR (95% CI)	*p* value
Male (male/female)		0.511 (0.234–1.115)	0.092		
BMI (${\mathrm{kg/m}}^{2}$)		0.884 (0.798–0.978)	0.017	0.889 (0.802–0.986)	0.026
Hypertension		6.097 (1.426–26.061)	0.015	5.577 (1.267–24.558)	0.023
CKD stage					
	no CKD/stage 1–2	Reference	-	Reference	-
	stage 3a–3b	2.475 (1.024–5.979)	0.044	1.997 (0.798–4.996)	0.139
	stage 4–5	8.250 (2.037–33.421)	0.003	6.729 (1.566–28.923)	0.010
${\mathrm{CHA}}_{2}$${\mathrm{DS}}_{2}$-Vasc		1.261 (0.972–1.636)	0.081		
HAS-BLED		1.465 (1.039–2.067)	0.030	1.184 (0.807–1.735)	0.387
Contrast volume		1.016 (1.000–1.032)	0.052		

AKI, acute kidney injury; LAAC, left atrial appendage closure; BMI, body mass index; CKD, chronic kidney disease.

### 3.4 AKI and Long-Term Outcome

**Fig. 3.**
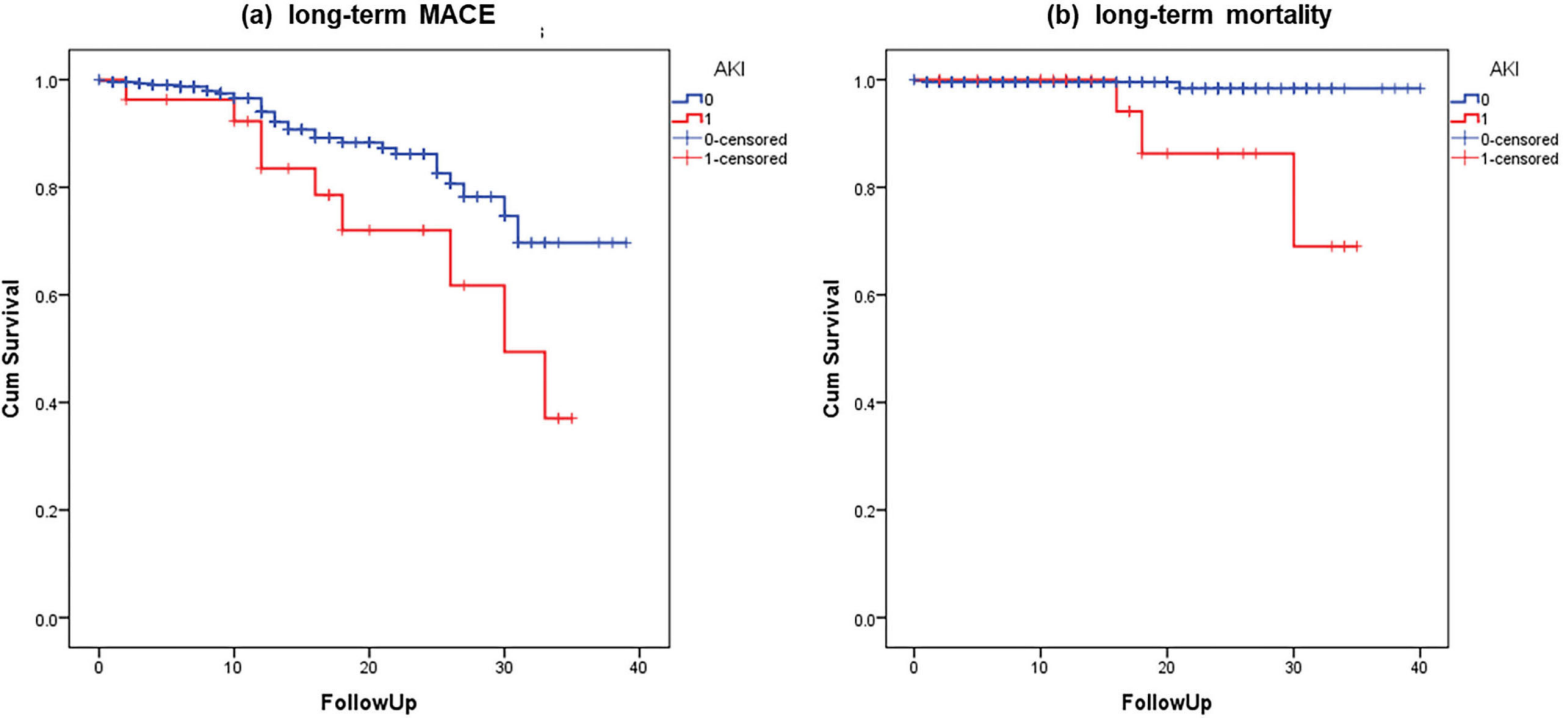
**Kaplan-Meier curves up to 3-year follow-up for (a) MACE between AKI vs. non-AKI, log rank = 0.017; (b) all-cause of mortality between AKI vs. non-AKI, log rank = 0.001**.

The median (IQR) follow-up time was 15 (4–26) months. The incidence of 1-, 2-, and 3-year MACE after the procedure was significantly higher in the AKI group than that in the non-AKI group (11.1% vs. 2.8%, *p* = 0.019; 18.5% vs. 3.7%, *p *< 0.001; 33.3% vs. 7.5%, *p *< 0.001). The hazard ratio (HR) for MACE showed an increasing trend over the years (Table 4). There was no statistically significant difference in the 1-year all-cause mortality between the two groups. The 2- and 3-year all-cause mortality rates after the procedure were significantly higher in the AKI group than that in the non-AKI group (7.4% vs. 0.7%, *p* = 0.001, and 11.1% vs. 0.9%, *p *< 0.001, respectively). The Kaplan–Meier estimate showed that long-term MACE (log rank, *p* = 0.017) and all-cause mortality (log rank, *p *= 0.001) were significantly higher in the AKI group than that in the non-AKI group (Fig. 3a,b).

**Table 4.** **Comparison of long-term outcomes between AKI vs. non-AKI group**.

		All	non-AKI group	AKI group	HR (95% CI)	*p* value
		n = 512	n = 485	n = 27		
MACE						
	1-year [n (%)]	15 (2.9%)	12 (2.5%)	3 (11.1%)	4.333 (1.146–16.391)	0.019
	2-year [n (%)]	21 (4.1%)	16 (3.3%)	5 (18.5%)	5.852 (1.964–17.440)	<0.001
	3-year [n (%)]	42 (8.2%)	32 (6.6%)	10 (37.0%)	6.203 (2.579–14.917)	<0.001
Mortality						
	1-year [n (%)]	2 (0.4%)	2 (0.4%)	0	0.940 (0.919–0.962)	0.722
	2-year [n (%)]	5 (1.0%)	3 (0.6%)	2 (7.4%)	11.333 (1.811–37.941)	0.001
	3-year [n (%)]	6 (1.2%)	3 (0.6%)	3 (11.1%)	13.250 (2.806–42.578)	<0.001

AKI, acute kidney injury; MACE, major adverse cardiac events.

## 4. Discussion

The principal findings of this study are as follows: (1) the incidence of AKI after LAAC in our center is relatively lower (5.3%), whereas the incidence of AKI in patients with renal insufficiency before LAAC is significantly higher (9.1% in CKD stage 3, 25% in CKD stages 4–5); (2) multivariate regression showed that low BMI, hypertension, and preoperative state of patients with CKD stages 4–5 are independent risk factors for AKI; (3) AKI after LAAC significantly increases the risk of midterm MACE and all-cause mortality, and the risk increases over the years.

The incidence of AKI ranges from 7% to 9.6% in patients undergoing PCI operation [1, 12, 13]. However, the incidence of AKI after interventional treatment for structural heart disease is much higher than that after PCI. For example, approximately 11.7% of patients develop AKI after TAVR [2], and 29% of patients after MitraClip [14] will develop AKI. Patients undergoing TAVR are usually older and frail with more comorbidities and more prone to hemodynamic disorders due to rapid cardiac pacing during the operation; therefore, the incidence of AKI is relatively higher than that in our study. Patients undergoing LAAC commonly have a venerable age, accompanied by various risk factors, such as hypertension, diabetes, and CKD. However, complications and hemodynamic instability were not observed during the LAAC procedure. Moreover, there is rare need for blood transfusion after LAAC. These may be the reasons for the lower incidence of AKI in LAAC patients than those who underwent TAVR and MitraClip.

In a previous study [5], the incidence of AKI after LAAC was 9%, whereas the overall incidence in our study was only 5.3%. Our study included much younger patients (69 years vs. 76 years), those with lower rates of hypertension (68.6% vs. 85.1%) and diabetes (21.9% vs. 33.2%) and lower rates of poor baseline renal function (CKD stages 4–5, 2.3% vs. 8.2%) than that of Nombela-Franco’s report, which may have contributed to the inconsistency of data. However, it is worth mentioning that 9% of LAAC operations in the study by Nombela-Franco *et al*. [5] were performed in conjunction with other operations, such as TAVR and PCI, which would increase the amount of contrast agent and theoretically increase the risk of contrast-induced kidney injury. We excluded patients who underwent combined surgery in our study. In another European multicenter study [6], the incidence of AKI after LAAC was as high as 13.7%. In this study, the subjects were older (75.1 years) and had a higher proportion of diabetes mellitus (34.7%) and CKD stages 4–5 (11.5%) at baseline. Furthermore, consistent outcomes were observed in that patients CKD stages 4–5 had the highest proportion of AKI (36.4% and 25%, respectively).

In the above two studies, the former found that the only independent predictor of AKI after LAAC was a poor baseline eGFR (HR, 1.32; 95% CI, 1.09–1.61; *p* = 0.004), and the latter revealed the worse renal function at baseline and a higher incidence of AKI and the CV/GFR ratio having a moderate predictive value for AKI (area under curve or AUC, 0.67; 95% CI, 0.50–0.84; *p* = 0.05). The CV/GFR ratio has been identified as an independent predictor of AKI, mainly in patients undergoing PCI [15]; however, in our study and Nombela-Franco’s study, no correlation was noted between the CV/GFR ratio and AKI. Contrary to our expectations, there was no significant correlation between the amount of contrast agent and the occurrence of AKI. This result is consistent with those of the two pioneering studies [5, 6]. Although the contrast volume in the AKI group had an increasing tendency compared with the non-AKI group, and the *p* value was very close to 0.05, it did not reach statistical significance. We would expand the sample size in further studies to confirm it. Our study suggests that low BMI, hypertension, and preoperative CKD stages 4–5 were independent risk factors for the occurrence of AKI.

Recently, a study evaluating the effect and safety of LAAC in patients with CKD and AF found that the incidence of AKI in the CKD group was 11.1%, which was significantly higher than that in the non-CKD group (0%) [16]. From the CRIC study, which is by far the largest prospective CKD cohort with nearly a decade of follow-up and systematic data collection, it is notable that incident atrial fibrillation was associated with a higher risk of developing end-stage renal disease (ESRD) in patients with CKD [17], and AKI has been recognized as a risk factor for CKD progression [18]. We previously reported [19] that the 2-year mortality and incidence of progressive CKD in patients with AKI after cardiac surgery significantly increased even after full recovery of renal function. A systematic review and meta-analysis [20] also identified AKI as an independent risk factor for CKD and ESRD. However, CKD is often considered a risk factor for AKI because of its epidemiological relationship [21]. Therefore, it is plausible to infer that patients with CKD and AF have poor prognosis after AKI.

At present, many preventive strategies for reducing the incidence of AKI have been reported, according to the recommendations for the prevention of contrast-induced nephropathy (CIN) [22], such as adequate hydration, use of low-osmolar or iso-osmolar contrast media, and minimal volume of contrast media. Adequate hydration remains the central component of CIN prevention, and this strategy should be implemented in patients with a low eGFR undergoing LAAC. A useful index to prevent CIN is the contrast volume to GFR ratio, which we strictly controlled below 3.7 in our daily practice to minimize the occurrence of AKI according to a previous study [15].

The innovation of LAA occlusion with decreased use of a contrast dye was recently described in a case report using the TrueFusion™ fusion-imaging system [23]. In turn, performing LAAC without contrast has been appealing, particularly in patients with severely reduced renal function. Sedaghat *et al*. [24] reported echocardiographically guided LAAC without the use of a contrast dye, and the technique appeared to be feasible without compromising the clinical effect and procedure safety. Thus, it is conceivable that completing LAAC with a reduced or no contrast agent is beneficial in special cases by decreasing AKI occurrence. The RenalGuard System has demonstrated benefits in reducing the occurrence of AKI in patients undergoing TAVR [25] and PCI [26], compared to hydration with normal saline solution, with a relative risk reduction of 79% and 74%, respectively. To the best of our knowledge, no standardized protocol for AKI prevention has been proposed for LAAC. The impact of strategies applied in PCI and TAVR on meaningful clinical outcomes in patients undergoing LAAC requires confirmation through larger and multicenter trials.

Surprisingly, these studies have consistently shown that the occurrence of AKI is related to in-hospital mortality, long-term mortality, and hospitalization due to renal and cardiac events [5, 6]. The proportion of noncardiac deaths was 68.3% in a report by Nombela-Franco. In our study, the 1-, 2-, and 3-year mortality rates were 0%, 7.6%, and 11.1%, respectively, in the AKI group, and the prognosis worsened over time. It is speculated that this poor condition is partially attributed to the deterioration of renal function after AKI.

## 5. Study Limitations

The main limitation of this study is its retrospective nature. Conversely, the incidence of AKI could be underestimated because we may have missed an AKI diagnosis after discharge. Finally, this was a single-center, observational study. Larger, multicenter studies are warranted to confirm these results.

## 6. Conclusions

Like other transcatheter interventions, it is very important to assess renal function before and after LAAC to facilitate early warning, identification, and intervention in patients who are prone to develop AKI. Reducing the risk of AKI may help improve the clinical outcomes of patients receiving LAAC. In patients with a high risk of deterioration of renal function, such as low BMI, hypertension, and preoperative CKD stages 4–5, further preventive measures and close monitoring should be undertaken after surgery.

## References

[1] James MT, Ghali WA, Knudtson ML, Ravani P, Tonelli M, Faris P (2011). Associations between acute kidney injury and cardiovascular and renal outcomes after coronary angiography. *Circulation*.

[2] Bagur R, Webb JG, Nietlispach F, Dumont E, De Larochelliere R, Doyle D (2010). Acute kidney injury following transcatheter aortic valve implantation: predictive factors, prognostic value, and comparison with surgical aortic valve replacement. *European Heart Journal*.

[3] Hindricks G, Potpara T, Dagres N, Arbelo E, Bax JJ, Blomström-Lundqvist C (2021). 2020 ESC Guidelines for the diagnosis and management of atrial fibrillation developed in collaboration with the European Association for Cardio-Thoracic Surgery (EACTS): The Task Force for the diagnosis and management of atrial fibrillation of the European Society of Cardiology (ESC) Developed with the special contribution of the European Heart Rhythm Association (EHRA) of the ESC. *European Heart Journal*.

[4] January CT, Wann LS, Calkins H, Chen LY, Cigarroa JE, Cleveland JC (2019). 2019 AHA/ACC/HRS Focused Update of the 2014 AHA/ACC/HRS Guideline for the Management of Patients With Atrial Fibrillation: A Report of the American College of Cardiology/American Heart Association Task Force on Clinical Practice Guidelines and the Heart Rhythm Society. *Journal of the American College of Cardiology*.

[5] Nombela-Franco L, Rodés-Cabau J, Cruz-Gonzalez I, Freixa X, Asmarats L, Gutiérrez H (2018). Incidence, Predictors, and Prognostic Value of Acute Kidney Injury among Patients Undergoing Left Atrial Appendage Closure. *JACC: Cardiovascular Interventions*.

[6] Sedaghat A, Vij V, Streit SR, Schrickel JW, Al-Kassou B, Nelles D (2020). Incidence, predictors, and relevance of acute kidney injury in patients undergoing left atrial appendage closure with Amplatzer occluders: a multicentre observational study. *Clinical Research in Cardiology*.

[7] James MT, Samuel SM, Manning MA, Tonelli M, Ghali WA, Faris P (2013). Contrast-Induced Acute Kidney Injury and Risk of Adverse Clinical Outcomes after Coronary Angiography. *Circulation: Cardiovascular Interventions*.

[8] Khwaja A (2012). KDIGO Clinical Practice Guidelines for Acute Kidney Injury. *Nephron Clinical Practice*.

[9] Inker LA, Astor BC, Fox CH, Isakova T, Lash JP, Peralta CA (2014). KDOQI us Commentary on the 2012 KDIGO Clinical Practice Guideline for the Evaluation and Management of CKD. *American Journal of Kidney Diseases*.

[10] Levey AS, Stevens LA, Schmid CH, Zhang YP, Castro AF, Feldman HI (2009). A New Equation to Estimate Glomerular Filtration Rate. *Annals of Internal Medicine*.

[11] Mehran R, Rao SV, Bhatt DL, Gibson CM, Caixeta A, Eikelboom J (2011). Standardized Bleeding Definitions for Cardiovascular Clinical Trials. *Circulation*.

[12] Amin AP, Bach RG, Caruso ML, Kennedy KF, Spertus JA (2017). Association of Variation in Contrast Volume with Acute Kidney Injury in Patients Undergoing Percutaneous Coronary Intervention. *JAMA Cardiology*.

[13] Tsai TT, Patel UD, Chang TI, Kennedy KF, Masoudi FA, Matheny ME (2014). Contemporary incidence, predictors, and outcomes of acute kidney injury in patients undergoing percutaneous coronary interventions: insights from the NCDR Cath-PCI registry. *JACC: Cardiovascular Interventions*.

[14] Tonchev I, Heberman D, Peretz A, Medvedovsky AT, Gotsman I, Rashi Y (2021). Acute kidney injury after MitraClip implantation in patients with severe mitral regurgitation. *Catheterization and Cardiovascular Interventions*.

[15] Laskey WK, Jenkins C, Selzer F, Marroquin OC, Wilensky RL, Glaser R (2007). Volume-to-creatinine clearance ratio: a pharmacokinetically based risk factor for prediction of early creatinine increase after percutaneous coronary intervention. *Journal of the American College of Cardiology*.

[16] Brockmeyer M, Wolff G, Krieger T, Lin Y, Karathanos A, Afzal S (2020). Kidney function stratified outcomes of percutaneous left atrial appendage occlusion in patients with atrial fibrillation and high bleeding risk. *Acta Cardiologica*.

[17] Bansal N, Xie D, Tao K, Chen J, Deo R, Horwitz E (2016). Atrial Fibrillation and Risk of ESRD in Adults with CKD. *Clinical Journal of the American Society of Nephrology*.

[18] Hannan M, Ansari S, Meza N, Anderson AH, Srivastava A, Waikar S (2021). Risk Factors for CKD Progression. *Clinical Journal of the American Society of Nephrology*.

[19] Xu JR, Zhu JM, Jiang J, Ding XQ, Fang Y, Shen B (2015). Risk Factors for Long-Term Mortality and Progressive Chronic Kidney Disease Associated With Acute Kidney Injury After Cardiac Surgery. *Medicine*.

[20] Coca SG, Singanamala S, Parikh CR (2012). Chronic kidney disease after acute kidney injury: a systematic review and meta-analysis. *Kidney International*.

[21] Waikar SS, Liu KD, Chertow GM (2008). Diagnosis, Epidemiology and Outcomes of Acute Kidney Injury. *Clinical Journal of the American Society of Nephrology*.

[22] Neumann FJ, Sousa-Uva M, Ahlsson A, Alfonso F, Banning AP, Benedetto U (2019). 2018 ESC/EACTS Guidelines on myocardial revascularization. *European Heart Journal*.

[23] Nelles D, Schrickel JW, Nickenig G, Sedaghat A (2020). Percutaneous left atrial appendage closure using the TrueFusion™ fusion-imaging technology. *Clinical Research in Cardiology*.

[24] Sedaghat A, Al-Kassou B, Vij V, Nelles D, Stuhr M, Schueler R (2019). Contrast-free, echocardiography-guided left atrial appendage occlusion (LAAo): a propensity-matched comparison with conventional LAAo using the AMPLATZER™ Amulet™ device. *Clinical Research in Cardiology*.

[25] Barbanti M, Gulino S, Capranzano P, Immè S, Sgroi C, Tamburino C (2015). Acute Kidney Injury with the RenalGuard System in Patients Undergoing Transcatheter Aortic Valve Replacement. *JACC: Cardiovascular Interventions*.

[26] Marenzi G, Ferrari C, Marana I, Assanelli E, De Metrio M, Teruzzi G (2012). Prevention of contrast nephropathy by furosemide with matched hydration: the MYTHOS (Induced Diuresis With Matched Hydration Compared to Standard Hydration for Contrast Induced Nephropathy Prevention) trial. *JACC: Cardiovascular Interventions*.

